# From the Road to the Field: Decoding Chemical Transformation in Aging Tire and Artificial Turf Crumb Rubber

**DOI:** 10.1021/acs.est.5c08260

**Published:** 2025-12-18

**Authors:** Madison H. McMinn, Yuqiao Tang, Phillip Berger, Katherine Poisson, Andresa Oliveira Tavares Lima, Aron Stubbins, Arzu Tuğçe Güler, Zhenyu Tian

**Affiliations:** a Department of Chemistry and Chemical Biology, College of Science, 1848 Northeastern University, Boston, Massachusetts 02115, United States; b Barnett Institute for Chemical and Biological Analysis, 1848 Northeastern University, Boston, Massachusetts 02115, United States; c Department of Bioinformatics, College of Science, 1848 Northeastern University, Boston, Massachusetts 02115, United States; d The Institute for Experiential A.I., Northeastern University, Boston, Massachusetts 02115, United States; e Department of Marine and Environmental Sciences, 1848 Northeastern University, Boston, Massachusetts 02115, United States; f Department of Civil and Environmental Engineering, Northeastern University, Boston, Massachusetts 02115, United States

**Keywords:** tire wear particles (TWP), artificial turf crumb rubber, cryomilled tire tread (CMTT), aging, 2,2,4-trimethyl-1,2-dihydroquinoline (TMQ), liquid chromatography−mass spectrometry (LC-MS), molecular networking, time-trend analysis, transformation products (TP)

## Abstract

Increasing tire use worldwide raises concerns about the health impact of rubber-derived chemicals. Of particular concern is the aging of these chemicals, which may lead to the formation of unknown, potentially harmful transformation products. Existing studies have not comprehensively assessed different aging mechanisms and their impact on rubber-derived chemicals from tires from various sources. To address current knowledge gaps, we exposed two tire and one artificial turf samples to outdoor and accelerated artificial photoaging, in dry and wet conditions over 12 weeks. Extracts underwent targeted quantitation, suspect, and nontarget screening via liquid chromatography–mass spectrometry followed by temporal trend analysis and molecular networking. Results indicate that accelerated photoaging and contact with water result in faster transformation/degradation rates. Temporal trend analysis prioritized 572 potential transformation products, including 37 proposed aging markers such as 1-phenylguanidine, which can guide future studies. Molecular networking revealed 180 potential 2,2,4-trimethyl-1,2-dihydroquinoline (TMQ)-related features, including two bioavailable compounds previously reported as para phenylbenzene diamines. We tentatively identified eight TMQ-related aging markers, including novel reaction products between TMQ and other rubber-derived chemicals. Our analysis highlights the complexity of tire and turf crumb rubber aging and the need for further investigation of rubber-derived chemicals and their transformation products.

## Graphical abstract

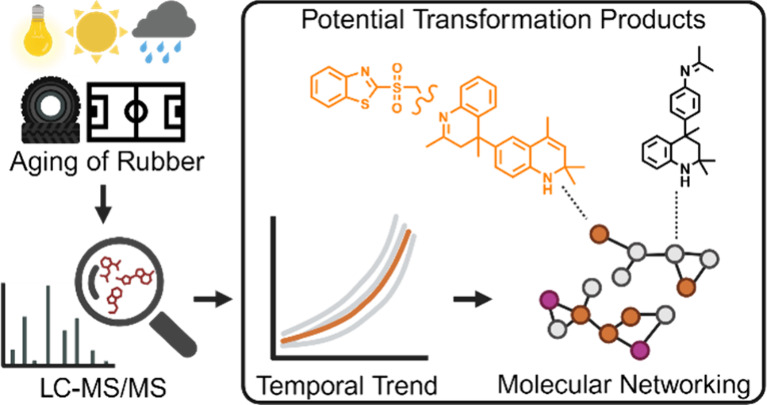

## 1 Introduction

The increasing use of tires across the globe raises concerns about potential environmental impacts. Approximately 3 billion new tires were produced, and 800 million entered the waste stream in 2019.^1^ Tire-derived particles and chemicals can enter the environment through various pathways, such as generation of tire road wear particles from active use tires,^2,3^ and the use of end-of-life tires as artificial turf infill.^4–6^ Of particular concern are the potential impacts of rubber-derived chemicals (RDCs) on human and environmental health, which have been detected in roadway runoff and roadway-impacted waterways,^7–10^ soil,^4,11,12^ air,^13,14^ and consumer products.^7^ Numerous RDCs have been found harmful for wildlife and the ecosystem^15–17^ such as the tire antiozonant *N*-(1,3-dimethylbutyl)-*N′*-phenyl-1,4-benzenediamine (6PPD) and its transformation product (TP), 6PPD-quinone, which is reported to cause acute mortality of aquatic species.^18,19^

Rubber toxicities can vary drastically due to aging and formation of TPs.^20–29^ To understand this variance in toxicity and the formation of TPs, recent studies have been conducted to gain insight into the changes to the chemical profile of RDCs under various aging conditions. However, current studies focused on RDCs^7,28,30^ or rubber leachate,^31–36^ and investigation of aging in tire rubber particles remains limited. In research focused on rubber particles, tire wear particles (TWPs) or cryomilled tire tread (CMTT) are typically utilized, with limited investigation of tire crumb rubber from artificial turf fields.^23,37^ Comparisons between the aging processes of tire crumb rubber and TWP/CMTT are crucial, as the variation in size and age of different rubber samples could impact aging mechanisms and rate.^23,38^ These studies found that photooxidation, ozonation, and thermal aging contributed to aging and the formation of TPs.^37,39–43^ However, the importance of these aging mechanisms is not fully elucidated, as evident by inconsistent reports discussing the impact of UV radiation.^37,43^ Additionally, limited studies suggest that artificial photoaging may not accurately reflect the aging process of natural conditions, leading to potentially inaccurate estimations of environmental toxicity and the degradation/transformation rate of RDCs.^37,43,44^ Preliminary research indicates that water may influence how tire crumb rubber ages, presenting an important area for further investigation.^42,43^ While there is agreement on trends of known RDCs, such as the decreasing trends of parent RDCs and increasing trends of known TPs, discrepancies are present in the rates of degradation/transformation.^37,43^ Additionally, studies of well-known parent compounds may not fully capture the chemical complexity of RDCs and could overlook unexpected or unknown compounds of interest and their interactions. To address current research gaps, we designed aging studies investigating a variety of tire samples under dry and wet conditions, subjected to natural and artificial photoaging.

To understand the chemical transformation process of rubber aging, we performed controlled aging experiments on rubber samples of different origins under both outdoor and accelerated photoaging in dry and wet conditions (Figure 1). We characterized their chemical profiles with targeted quantitation, suspect screening, and nontarget screening (NTS), using liquid chromatography-tandem mass spectrometry (LC-MS/MS). Targeted analyses quantified known compounds,^37,43^ and NTS identified other potential TPs.^8,9,45,46^ For features detected from NTS, trend-based clustering aided in prioritization of potential TPs.^47–49^ After trend-based prioritization was performed, molecular networking was applied to identify potential TPs. This approach was applied to assign potential identities to unknowns based upon their clustering with known compounds based on fragmentation/structural similarity.^50–56^ Integrating these approaches, aging markers of RDCs were assessed to provide insight into potential human and environmental toxicity and inform proper recycling/management of end-of-life tires.

**Figure 1:**
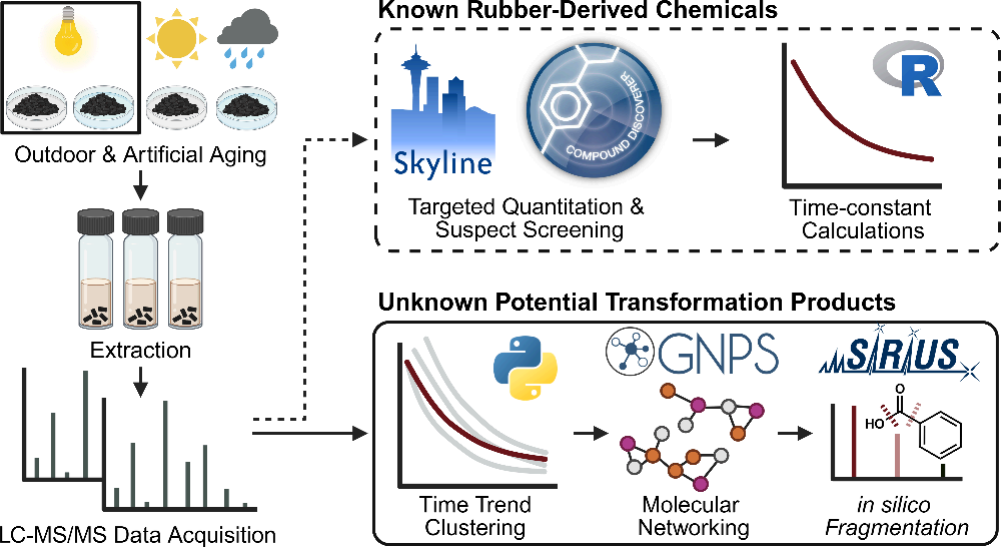
Workflow of sample preparation, data acquisition, and the data analysis approach.

## 2 Materials and Methods

### 2.1 Materials

RDC standards and isotopically labeled internal standards (ISTDs) were purchased commercially (Table S1). OPTIMA grade water, methanol, acetone, formic acid, and ammonium fluoride were purchased from Fisher Scientific (Pittsburgh, PA, USA). Artificial turf crumb rubber (*n* = 1) was collected in dry weather (>48 h prior dry weather period) from 3 points (end goals and midline) of a new artificial turf field in New Hampshire, USA. Two representative tire samples were used for experiments: a tire wear particle mixture of 2 new and 7 used tires via physical abrasion^8^ (TWP) and a cryomilled tire tread mixture^57^ (CMTT, Table S2) of 12 new tires provided by the US Tire Manufacturers Association. Prior to extraction, samples were stored in the dark at 4 °C.

### 2.2 Outdoor Aging

Artificial turf and tire samples were aged on the roof of a three-story building on Northeastern University’s campus (42.34067, −71.09171) for 12 weeks from May 21, 2024 to August 13, 2024. Rubber (2.4 g) was weighed into solvent cleaned 100 mm × 15 mm glass Petri dishes covered with quartz lids to prevent sample loss, with two Petri dishes for each rubber sample type (one in dry conditions and one in wet conditions, Figure S1) resulting in a total of six Petri dishes. To simulate outdoor aging, in which samples were exposed to rainwater, a slightly modified aging procedure was conducted for the wet samples. Wet samples started with 30 mL of Milli-Q water, with up to 30 mL of rainwater (the maximum volume each Petri dish could hold) added throughout aging based on weather (Table S3). Rainwater was collected via nonsterile filtration with a stainless steel mesh and 27 mm type D28 cellulose filters into a 32 oz glass jar placed next to the samples during rain events. Sampling occurred at 1, 2, 4, 8, and 12 weeks, in which 0.4 g of rubber was collected from each dish and stored in the dark at 4 °C until extraction. If rainwater was present in the wet samples at the time of collection, samples were filtered to dryness before collection. After filtration, 0.4 g of sample was collected from each dish, and the remainder was returned to the dish for further aging until the next time point.

### 2.3 Accelerated Photochemical Aging

Accelerated photochemical aging was performed in a SUNTEST XLS+ weathering chamber (Atlas Material Testing Solutions, USA) equipped with one 1700 W xenon lamp with a daylight filter operated with an irradiance of 765 W/m^2^ (300–800 nm). Conversion between accelerated photochemical aging time and natural days was calculated based on the annual global horizontal irradiance of 148 kW/m^2^ in Boston, MA (42.36066, −71.05791).^58^ Rubber (0.125 g) was weighed into Petri dishes covered with quartz lids for dry conditions or 250 mL round-bottom flasks containing 125 mL of Milli-Q water for wet conditions. Samples were aged for 1.5, 6, and 18 days, which corresponds to 1, 4, and 12 weeks of sunlight exposure, respectively, corresponding to an accelerated aging factor of approximately 4.6. The crumb rubber samples were collected and stored in amber vials at 4 °C until extraction.

### 2.4 Sample Extraction

Rubber samples (50 mg) were extracted in 5 mL of 1:1 methanol:acetone (v:v) in duplicate.^59^ Samples were sonicated for 1 min followed by agitation for 24 h at room temperature in the dark. After extraction, samples were syringe filtered using a 0.22 μm PTFE filter into LC vials and stored at −20 °C until analysis. Alongside real samples, duplicate method blanks were prepared using empty vials following the same preparation workflow as that for the samples.

### 2.5 Targeted Quantitation

Extracts were analyzed using an Agilent 1200 liquid chromatograph interfaced to a Thermo Fisher TSQ Altis triple quadrupole mass spectrometer equipped with a heated electrospray ionization source (HESI). Chromatographic separation was performed using a C18 column (Agilent InfinityLab Poroshell 120 EC-C18, 2.1 mm × 50 mm, 2.7 μm). Method details including instrument parameters (Table S4) and targeted MS/MS transitions (Table S5) are present in the Supporting Information. Solvent blanks and ISTD controls were analyzed every 15 samples. The isotope-dilution method was utilized to compensate for matrix effects and recovery during sample preparation.^60,61^ The retention time (RT) variations of ISTD mixture components were ± 0.1 min across all samples. Data analysis was performed in Skyline Daily ver. 24.1.1.449 ^62^ with settings in the Supporting Information (Table S6). Ten-point calibration curves were prepared using a standard mixture ranging from 0.05 to 1000 ng/mL (0.05, 0.1, 1, 5, 10, 50, 100, 250, 500, and 1000 ng/mL) spiked with ISTDs at 50 ng/mL. Sample concentrations were adjusted to account for background by subtracting method blank concentrations. Analytical figures of merit including *R* ^2^, limit of detection, limit of quantitation, interday precision, and relative and absolute recovery were assessed in accordance with previous studies (Table S7).^59^

### 2.6 Nontarget and Suspect Screening

Crumb rubber extracts were analyzed by using a Thermo Fisher Scientific Vanquish Flex ultrahigh-performance liquid chromatograph interfaced to a Thermo Fisher Scientific Exploris 240 quadrupole-orbitrap mass spectrometer equipped with an HESI source. Chromatographic separation was performed using a C18 column (Agilent ZORBAX RRHD Eclipse Plus C18, 2.1 mm × 100 mm, 1.8 μm). Method details are present in the Supporting Information (Table S8). Solvent blanks and ISTD controls were analyzed every 15 samples. The RT variations of ISTD components were ±0.1 min, and precursor mass errors were <3 ppm across all samples. Spectral preprocessing including peak picking, retention time alignment, compound grouping, library, and mass list searches were done in Compound Discoverer v3.3 (Thermo Fisher Scientific, Table S9). Features with average peak area 3X higher in at least one sample group compared to the average blank were kept for further analysis. For NTS and temporal trend-based clustering, a further restriction of 50% CV for the artificial turf crumb rubber sample and 30% CV for the TWP and CMTT samples was added. Compounds underwent molecular networking using GNPS (Text S1, Table S10), and Cytoscape was used for visualization.^63,64^ SIRIUS was used for *in silico* structural identification (Table S11).^65^ Identification confidence was assigned based on the Schymanski levels.^66^ The nontarget screening study reporting tool developed by Peter et al.^67^ was used in the preparation of this manuscript, and NORMAN guidance^68^ was followed.

### 2.7 Kinetic Modeling and Unsupervised Temporal Trend Clustering

Kinetic modeling was applied to analyze the chemical degradation patterns under various environmental conditions. Our approach follows established methodologies for time–concentration profile analysis adapted from tire particle degradation studies and was performed in R ver. 4.4.2 (Text S2).^43^ Unsupervised temporal trend clustering of NTS data was performed in Python ver. 3.9 using hierarchical density-based spatial clustering of applications with noise (HDBSCAN, Text S3).^69^

## 3 Results and Discussion

### 3.1 Aging Conditions Impact Rates of Formation of Rubber-Derived Chemicals

#### 3.1.1 Target and Suspect Screening Results

Concentrations of 27 RDCs extracted from tire and artificial turf crumb rubber samples in wet and dry conditions were quantified at different time points of outdoor or photochemical aging (Table S12). Besides targeted quantitation data, we used suspect screening to broaden our compound investigation. In total, 80 compounds were identified (Figure 2 and Table S13) and categorized into one of six groups based on their purpose in the crumb rubber manufacturing process: vulcanization accelerators (30%), vulcanization additives (16%), plasticizers and surfactants (19%), transformation products (18%), antioxidants (14%), and benzanilides (4%).^70^ For these 80 compounds, 25 were identified as level 1, 43 as level 2, 8 as level 3, and 4 as level 4.

**Figure 2:**
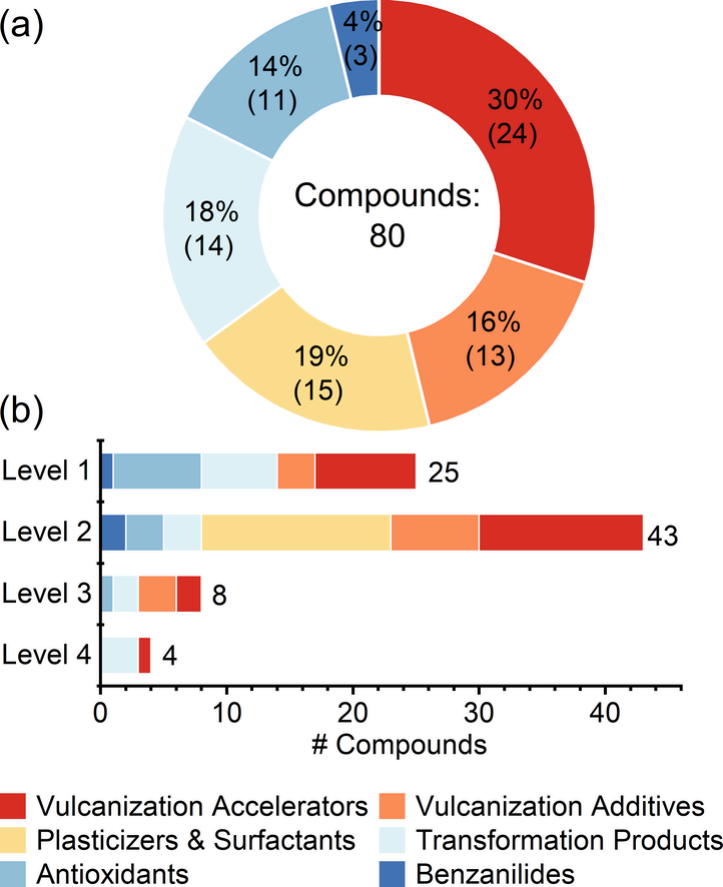
Rubber-derived chemicals identified from target and suspect screening.

#### 3.1.2 Trends of Rubber-Derived Chemicals

The kinetics of degradation/formation were assessed for all conditions through the calculation of a time-constant (τ), and kinetic modeling was only achieved when at least 3 time points contained concentrations above the limit of quantitation (Table S14). Otherwise, kinetic modeling was performed from suspect screening results (Table S15). Based on the irradiance strength of the solar simulator, the acceleration factor under photoaging should be approximately 4.6 times faster than outdoor aging conditions under direct sunlight at the Earth’s surface. However, when comparing the rate of outdoor and accelerated photoaging under the same conditions, most chemicals undergo degradation at a faster rate than predicted (median ratio, τ_photo_/τ_outdoor_: dry turf: 10, wet turf: 38, dry TWP: 30, wet TWP: 30, dry CMTT: 21, wet CMTT: 25, Table S15). The observed difference suggests while photoaging contributes to rubber chemical degradation,^28,31,34^ other factors like temperature fluctuations, dry-wet cycles, and rainwater composition may explain variations in aging kinetics between outdoor and accelerated conditions. Additionally, variation in the incident radiation reaching particles at the bottom of the Petri dish/round-bottom flask aged under wet conditions may lead to discrepancies in the aging kinetics as well. Outdoor aging experiments were performed in Petri dishes where water was added to a maximum depth of ∼13 mm, whereas accelerated aging was performed in round-bottom flasks where water was added to a depth of ∼35 mm. Differences in the depth of particles at the bottom of these two different containers could impact the incident radiation received by particles at the bottom of each container. However, in both the Petri dishes and round-bottom flasks, rubber particles primarily floated at the surface of the water, due to their hydrophobic properties, so the amount of particles impacted by the difference in water depth may be minimal.^23^ Degradation trends under wet, outdoor aging conditions reported by Fohet et al.^43^ for TMQ, 6PPD, DPG, and benzothiazole are similar to those observed in this study for CMTT and TWPs aged via outdoor wet conditions. Additionally, degradation trends under dry, accelerated artificial aging conditions reported by Fohet et al.^43^ for TMQ and 6PPD are similar to those found in this study for CMTT and TWPs aged under dry artificial photoaging conditions. However, degradation trends for DPG and benzothiazole in the CMTT and TWPs aged under dry artificial photoaging conditions in this study differ from those reported by Fohet et al.^43^ under similar aging conditions. When comparing aging rates between crumb rubber samples aged outdoor under dry and wet conditions, we observed that water accelerated the rate of chemical degradation/transformation (Table 1, median ratio τ_outdoor_ dry/wet turf: 2.3, TWP: 1.3, CMTT: 1.6). Although rubber aging has been studied,^42,43^ the impact of water in outdoor aging conditions is often overlooked. Because water can affect aging rates and potentially reactions, studying tire crumb rubber under dry conditions alone may not reflect real environmental conditions. Therefore, we focus our discussion on CMTT, TWP, and turf crumb rubber aged outdoors with rainwater exposure.

**Table 1:** Time Constants (τ) of Cryomilled Tire Tread (CMTT), Tire Wear Particles (TWP), and Artificial Turf Crumb Rubber (Turf) Samples under Outdoor, Wet Aging Conditions

		**CMTT**			**TWP**			**Turf**		
**compound**	**trend**	**dry**	**wet**	dry/wet	**dry**	**wet**	dry/wet	**dry**	**wet**	dry/wet
DPG	D	173 ± 41	103 ± 19	1.7	^b^	^b^		30 ± 11	^b^	
BTH	D	^b^	8 ± 2		^b^	11 ± 3		^b^	4 ± 2 (N)	
TMQ	D	16 ± 1	5 ± 0.09 (N)	3.2	53 ± 6	4 ± 0.1 (N)	13	26 ± 8	4 ± 1 (N)	6.5
6PPD	D	31 ± 3	22 ± 2	1.4	80 ± 6	51 ± 10	1.6	49 ± 9	24 ± 4	2.0
6QDI	D	43 ± 28 (I)	^b^		18 ± 3	16 ± 2	1.1	^b^	^b^	
6PPDQ	I	20 ± 2.5	^b^		77 ± 25	59 ± 13	3.1	^b^	^b^	
MBT	D	11 ± 1	8 ± 1	1.4	15 ± 1	11 ± 2	1.4	70 ± 21	45 ± 10	1.6
2-amino-BTH	I	41 ± 7	21 ± 11	2.0	61 ± 14	59 ± 13	1.0	^b^	^b^	
HMMM	D	36 ± 4	27 ± 2	1.3	79 ± 10	26 ± 6	3.0	^b^	^b^	
benzanilide	I	62 ± 40	^a^		46 ± 7	^b^		^b^	^b^	

a Compound not detected in the sample.

b Compound detected; trend could not be fitted.

Under wet outdoor aging conditions, we observed 14 compounds following a decreasing trend in all three sample types, including 6PPD, MBT, TMQ, and BTH, suggesting continued transformation in tire and artificial turf crumb rubber during a period of 12 weeks. In the turf crumb rubber sample, we observed four compounds with an increasing trend, only one of which was an expected TP (TP 280b). In the TWPs, three compounds were found to increase, including 2-amino-BTH, which has been proposed as a possible TP.^59^ The CMTT sample had the highest number of increasing compounds (14) including 2-amino-BTH, 2-SO_3_-BTH, and PPD-related TPs, including DPPD-quinone, 6PPD-quinone, DTPD-quinone, TP 256, TP 274, and TP 280b. Comparison of aging kinetics between turf crumb rubber and CMTT/TWPs indicates that turf crumb rubber degradation/transformation is faster than TWPs but slower than CMTT (median ratio τ_outdoor_ wet: turf/CMTT: 1.3; turf/TWP: 0.95). However, the wide range of these values indicates that this observation may be compound-specific (range of τ_outdoor_ wet: turf/CMTT: 0.012–6.0; turf/TWP: 0.14–5.0). We suggest future studies to include turf crumb rubber alongside CMTT or TWP and compare materials from different tire manufacturers to assess crumb rubber’s chemical composition and aging processes more comprehensively. Additionally, we observed a continued decrease in known compounds, such as 6PPD, without a corresponding increase in the number of known TPs. This suggests the formation of many unknown TPs or loss of parent compound to terminal products of photodegradation.

### 3.2 Temporal Trend Reinforced Molecular Networking Prioritizes Potential TPs in Tire Crumb Rubber Samples

#### 3.2.1 Unsupervised Cluster Analysis Prioritizes Potential TPs

Only ∼4–5% of the features present in the crumb rubber samples were identified from suspect screening (CMTT: 52/1,152, TWP: 52/1020, turf crumb rubber: 27/651). This implies a great level of chemical complexity that warrants further characterization of the chemical profile of these samples. To prioritize unknown TPs, we performed an unsupervised clustering analysis (HDBSCAN) to group features. Initial HDBSCAN clustering yielded three major clusters (noise cluster −1, cluster 0, and cluster 1). Cluster 1 contained features with different systematic temporal trends, so it was further subclustered using K-means (*k* = 4) for refinement based on temporal similarity, yielding four subclusters (clusters 10–13, Text S3). Among the total six clusters (−1, 0, 10, 11, 12, and 13) in the CMTT sample, clusters 0 exhibited an increasing trend, clusters 11 and 12 showed intermediate patterns, clusters 10 and 13 showed decreasing trend, and cluster −1 had no clear trend (noise). Thus, these six clusters displayed three temporal trends that were meaningful in chemical transformation context: increasing, intermediate, and decreasing (Figure 3 and Figures S2 and S3), in addition to features without a clear trend. In the CMTT sample, 16% of features followed an increasing trend, 20% intermediate, and 29% decreased (Table S16). In the TWP samples, we observed 18% of features following an intermediate trend and 58% decreasing, with no cluster of only increasing trends found (Table S17). In the turf crumb rubber, 8% of features followed an increasing trend and 26% decreasing. There were no intermediate features found (Table S18). Unknown features following an increasing or intermediate trend were prioritized as potential aging TPs. CMTT had the highest % of potential TPs (36%), followed by TWP (18%), and then turf crumb rubber (8%). This trend is expected; as the crumb rubber samples increase in age, they generate less TPs. Among the 3 crumb rubber samples, we found 572 potential TPs (Table S19). In addition, 20 TPs were found in all samples, and 37 were found in CMTT and turf crumb rubber but not TWPs (Table 2 and Table S20). Due to the composition of the TWP sample (mixture of 9 new and aged tires) and the fact that no increasing cluster was found, we prioritized the 37 features found in CMTT and turf as aging markers. The increasing and/or intermediate trends in the samples (CMTT, TWP, and crumb) suggest that chemical transformation is a long-term process, and some TPs can be stable/persistent.

**Figure 3:**
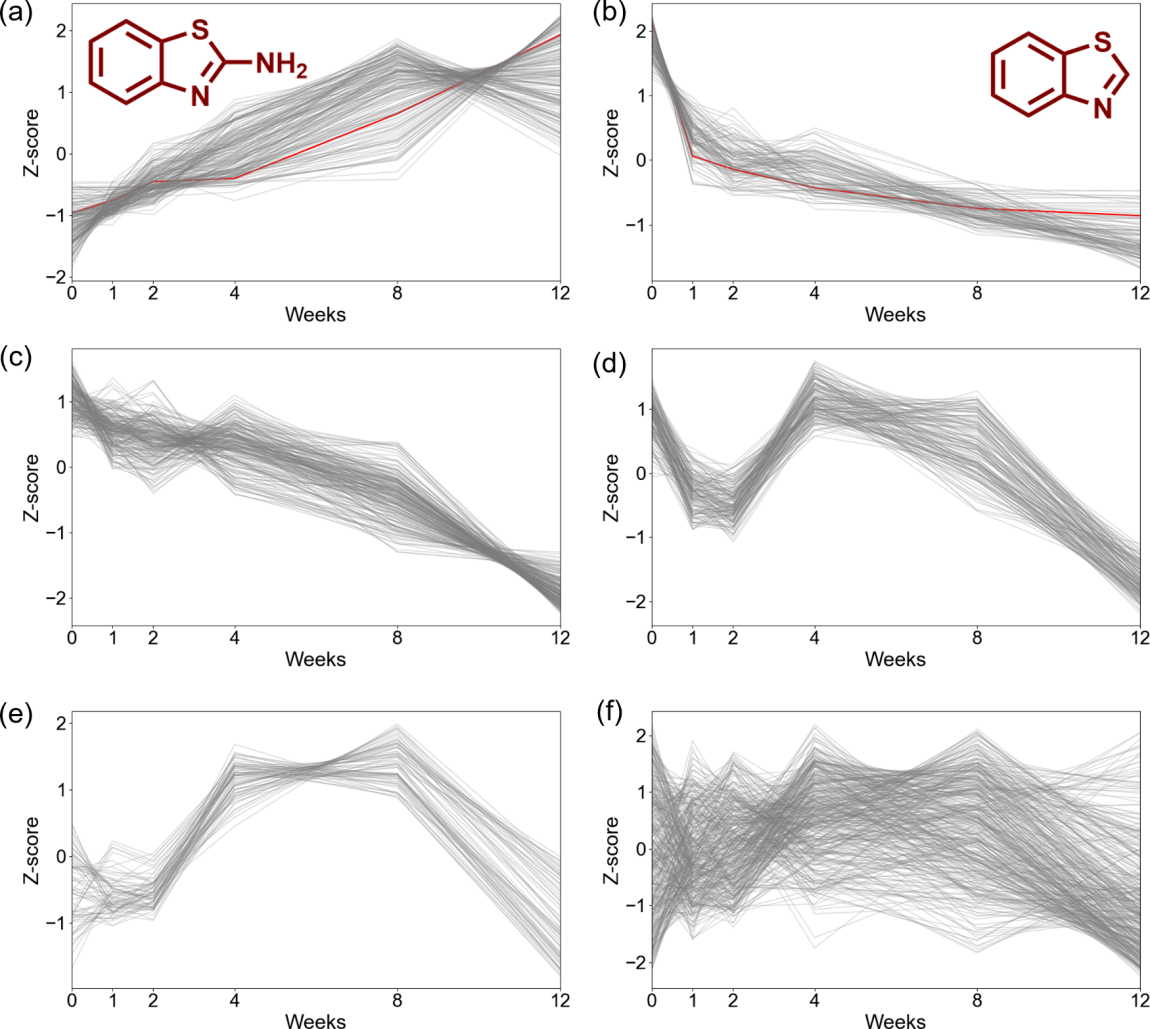
Unsupervised temporal trend clusters generated by HDBSCAN (followed by K-means for cluster 1) for naturally aged CMTT wet samples for cluster # (a) 0, increasing with red line indicating 2-amino-benzothiazole; (b) 10, decreasing with red line indicating benzothiazole; (c) 13, decreasing; (d) 11, intermediate; (e) 12, intermediate; and (f) −1, no trend.

**Table 2:** Aging Markers in CMTT, TWP, and Turf Crumb Rubber (Additional Information Is Present in the Supporting Information, Table S20)

ID #	**name**	**formula**	**calc. MW**	*m*/*z*	**RT (min)**	**ID level**
TP-1		C_7_H_6_N_2_O	134.0479	135.0552	3.3	4
TP-2	1-phenylguanidine	C_7_H_9_N_3_	135.0796	136.0868	1.8	2a
TP-3	2-(hexylamino)acetic acid	C_8_H_17_NO_2_	159.1258	160.1331	3.6	3
TP-4^a^	TMQ-C11H11NO	C_11_H_11_NO	173.084	174.0913	7.4	3
TP-5		C_14_H_15_N	197.1204	198.1276	8.2	4
TP-6		C_13_H_13_NO	199.0996	200.1069	4.3	4
TP-7^a^	4-(aminomethyl)-*N*-cyclohexylaniline	C_13_H_20_N_2_	204.1625	205.1698	7.9	3
TP-8	hexylpyridine-2-carboxylate	C_12_H_17_NO_2_	207.1258	208.1331	9.7	3
TP-9	*N*-phenyl-1*H*-benzimidazol-2-amine	C_13_H_11_N_3_	209.0952	210.1025	6.2	3
TP-10^b^		C_14_H_17_NO	215.1309	216.1382	4.6	4
TP-11		C_14_H_12_N_2_O	224.0949	225.1022	16.8	4
TP-12^a^	benzothiazol-2-yl-*o*-tolyl-amine	C_14_H_12_N_2_S	240.0721	241.0794	18.1	3
TP-13^a^	(1*E*,2*E*)-2-(2*H*-benzo[*b*][1,4]oxazin-2-ylidene)-*N*-(2-methylpentyl)ethan-1-imine	C_16_H_20_N_2_O	256.1575	257.1648	22.6	3
TP-14^a^	(1*Z*,2*E*,4*E*)-4-((2-methylpentyl)imino)-1-(phenylimino)but-2-en-2-ol	C_16_H_22_N_2_O	258.1731	259.1804	20.7	3
TP-15		C_16_H_22_N_2_O_2_	274.168	275.1753	8.4	4
TP-16	7-(*sec*-butylamino)-2,3,4,4*a*,10,10*a*-hexahydro-1*H*-phenoxazin-1-ol	C_16_H_24_N_2_O_2_	276.1836	277.1909	7.7	3
TP-17	(*E*)-4-methyl-*N*-(1*H*-phenothiazin-7-yl)pentan-2-imine	C_18_H_20_N_2_S	296.1346	297.1419	22.9	3
TP-18^a^ ^,^ ^b^		C_17_H_20_N_6_	308.1752	309.1824	20.7	4
TP-19^a^		C_16_H_12_N_3_O_2_P	309.0676	310.0748	16.3	4
TP-20		C_18_H_20_N_2_OS	312.1295	313.1368	16.6	4
TP-21^a^ ^,^ ^b^		C_13_H_15_N_6_PS	318.0825	319.0898	22.5	4
TP-22		C_21_H_22_N_2_O	318.173	319.1803	10.0	4
TP-23^a^	2,2,2′,3′,4,4′-hexamethyl-1,2,3,4-tetrahydro-4,6′-biquinoline (TMQ-C_24_H_28_N_2_)	C_24_H_28_N_2_	344.225	345.2323	12.9	3
TP-24^a^	TMQ-C_20_H_28_N_4_	C_20_H_28_N_4_	324.2298	347.2191	19.2	3
TP-25^a^	TMQ-C_17_H_34_N_2_OS_2_	C_17_H_34_N_2_OS_2_	346.212	347.2192	19.2	3
TP-26		C_20_H_30_N_6_	354.2533	355.2605	25.0	4
TP-27		C_22_H_19_N_3_O_2_	357.1476	358.1548	16.3	4
TP-28^a^ ^,^ ^b^		C_19_H_18_N_2_O_2_S_2_	370.0808	371.088	25.7	4
TP-29		C_26_H_30_N_2_O	386.2356	387.2429	12.9	4
TP-30^a^		C_25_H_27_N_3_S	401.1923	402.1996	26.6	4
TP-31^a^		C_30_H_30_N_4_	446.2468	447.2541	19.4	4
TP-32^b^		C_29_H_48_N_2_O_4_	488.3631	489.3703	29.3	4
TP-33^a^	TMQ-C_36_H_43_N_3_	C_36_H_43_N_3_	517.3456	518.3529	16.6	3
TP-34	TMQ-C_30_H_29_N_3_O_2_S_2_	C_30_H_29_N_3_O_2_S_2_	527.17	528.1773	18.3	3
TP-35^a^	TMQ-C_31_H_33_N_3_O_2_S_2_ isomer	C_31_H_33_N_3_O_2_S_2_	543.2012	544.2085	25.6	3
TP-36^a^	TMQ-C_31_H_33_N_3_O_2_S_2_ isomer	C_31_H_33_N_3_O_2_S_2_	543.2014	544.2087	22.7	3
TP-37^a^		C_29_H_29_N_9_S_2_	567.2014	568.2087	20.8	4

a Only in CMTT and turf crumb rubber.

b Poor MS/MS.

#### 3.2.2 Integrating Temporal Trends and Molecular Networking for Identification of Potential Transformation Products

After the potential TPs were prioritized based on their temporal trend (increasing or intermediate) within samples, molecular networking was employed to aid in structural identification through similarity analysis. By performing temporal trend analysis prior to molecular networking, network clusters were employed to assess what compounds were structurally similar to the 572 potential TPs, by grouping chemical features based on MS/MS similarity. When annotating potential TPs using molecular networking, we utilized compounds identified/annotated during suspect screening (Figure 4, pink squares) that were present within the same cluster as potential TPs to annotate TPs to a class of known RDCs based on their MS/MS similarity within each cluster (Figure 4, orange circles). By utilizing this approach, we assigned unknown TPs to a broad class of RDCs without proposing direct precursor-TP pairs. Utilizing this approach, we were able to identify/annotate 63 potential TPs related to known RDCs 6PPD, benzothiazole, 1,3-dipehynlguanidine (DPG), hexamethoxymethylmelamine (HMMM),^71^ and TMQ, including 45 that were not reported previously (Table S21). DPG and it's suspected TP, *N*,*N′*-diphenylurea^72,73^ were prioritized as potential TPs, along with 12 DPG-related compounds and six *N*,*N′*-diphenylurea-related compounds. Seven of these, including oxidized DPG-OH isomers, *N*-cyclohexyl-*N*′-phenylguanidine, 1,2-diphenyl-3-cyclohexylguanidine, and *N*,*N*″-bis­[2-(propan-2-yl)­phenyl]-guanidine, were previously reported in road dust samples, but this is the first time they are proposed as potential TPs.^24^ Additionally, 2 of these compounds were prioritized in the 37 aging markers, including 4-(aminomethyl)-*N*-cyclohexylaniline (TP-7) and 1-phenylguanidine (TP-2, Table 2).

**Figure 4:**
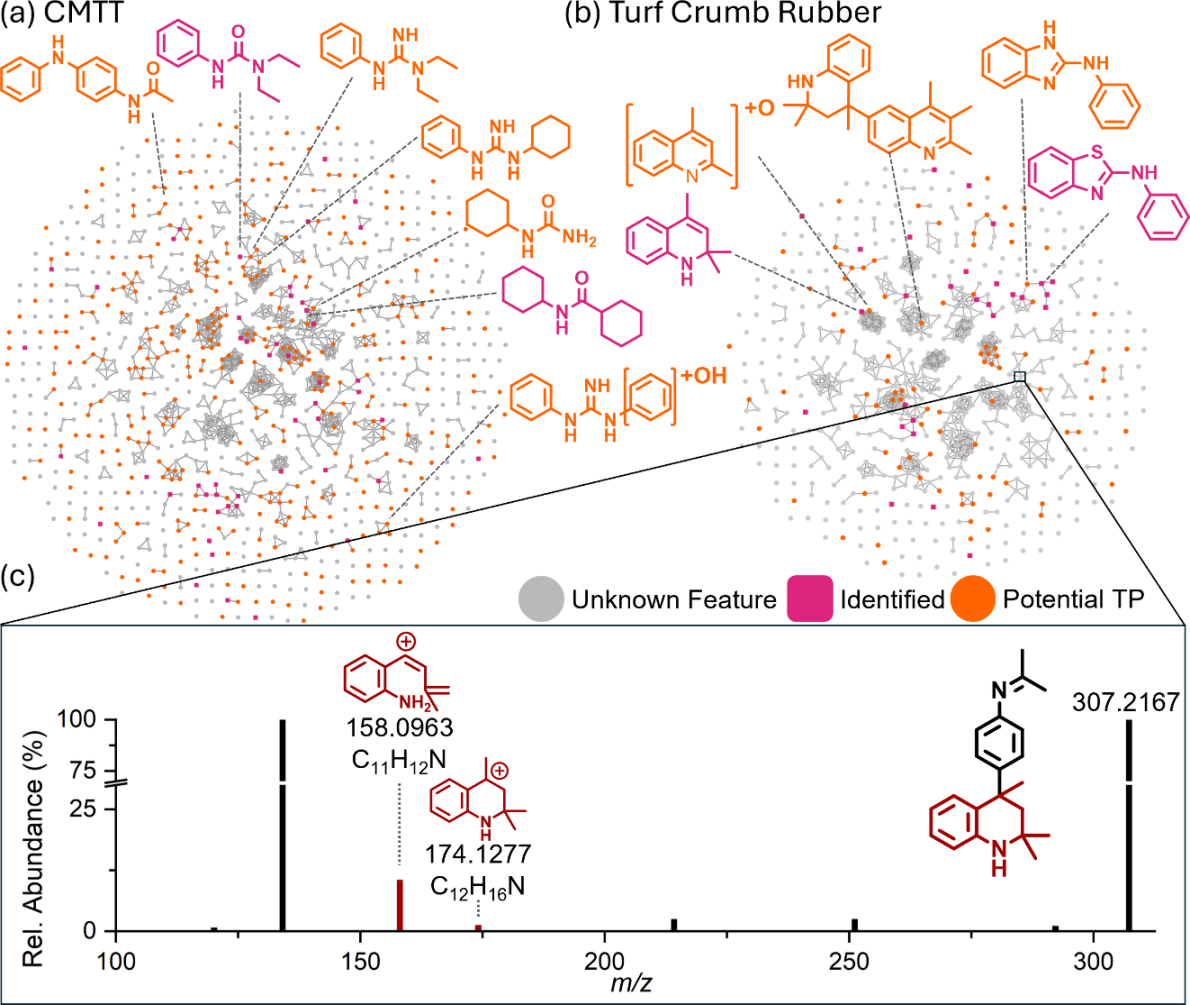
Molecular networks of all NTS features in (a) cryomilled tire tread (CMTT) and (b) turf crumb rubber with identified compounds from suspect screening in pink and potential TPs in orange (c) MS/MS of TMQ-C_21_H_26_N_2_ with TMQ diagnostic fragments in dark red.

We also annotated 13 potential PPD-related TPs, including 6PPD-quinone. Twelve of the potential PPD-related TPs are likely results of oxidation, in line with previous studies.^2,18,35,55,74^ Additionally, we identified three TPs related to *N*-(4-anilinophenyl)­formamide, including one new potential TP, *N*-(4-anilinophenyl)­hexanamide.^24^ These PPD TPs are related to *N*-(4-anilinophenyl)­formamide (PPD-CHO) and were previously found in road dust but were not confirmed as TPs.^24^ While PPD-CHO was detected in all three samples, it was found to have no trend in CMTT, and a decreasing trend in the TWP and turf crumb rubber samples, suggesting that it may be formed during the rubber manufacturing process. Three PPD-related TPs were prioritized in the 37 aging markers, including two that have been previously annotated (TP-13, TP-14).^35^

#### 3.2.3 Application of Integrated Prioritization to Elucidate Selected Network Clusters

Each sample contained a major cluster (second largest in turf crumb rubber and TWP, third in CMTT) with many nodes of unknown compounds, which may be potential TPs but no identified or annotated compounds, suggesting a group of structurally related compounds that were not closely related to any compound within the known compound library used for network analysis. Further investigation of MS/MS spectra for these compounds revealed common fragments of *m*/*z* 174.1276, 159.1040, 158.0967, 132.0806, 118.0649, and 106.0653 (Figure 4c, Figure S4, and Table S22), which suggested that they were related to tire antioxidant, TMQ (2,2,4-trimethyl-1,2-dihydroquinoline). While monomeric TMQ is typically utilized in targeted analysis of rubber-derived materials, TMQ is added during the rubber manufacturing process in a polymeric form and may produce TMQ oligomers in rubber materials (Table S18).^75–80^ We tentatively identified two TMQ-oligomeric compounds, *N*-(4-(2,2,4-trimethyl-1,2,3,4-tetrahydroquinolin-4-yl)­phenyl)­propan-2-imine (TMQ-C23H26N2, *m*/*z* 331.2168, Figure S5b) and 2,2,2′,4,4′-pentamethyl-1,2,3,4-tetrahydro-4,6′-biquinoline (TMQ-C21H26N2, *m*/*z* 307.2167, Figure 4c and Figure S6b). Identification of these two TMQ-oligomeric compounds was further confirmed by the analysis of a commercially available poly-TMQ standard (AmBeed, Catalog #A615697, Figures S5a and Figure S6a). A previous lumpfish exposure study prioritized two compounds with the same formulas (C_23_H_26_N_2_ and C_21_H_26_N_2_) as the most bioavailable rubber contaminants.^81^ While the authors suspected that the two unknowns are PPD-derivatives, MS/MS fragments *m*/*z* 174.1276 and 158.0967 indicated their correlation with TMQ. By comparing spectra from the poly-TMQ standard, rubber samples, and from Hagg et al. (Figure S6c),^81^ we propose that these bioavailable compounds may be TMQ oligomers, instead of PPD-derivatives as previously reported. However, due to differences in the instrumentation utilized herein (LC-MS equipped with an electrospray ionization source) and that of Hagg et al.^81^ (GC-MS equipped with an electron ionization source), direct comparison of the MS/MS spectra is challenging. As a result, we can only classify the compounds reported by Hagg et al.^81^ as potential TMQ oligomers without additional analysis using similar instrumentation to that of this study. These bioavailable potential TMQ oligomers warrant further investigation on their occurrence and potential toxicity.

The presence of TMQ-oligomeric compounds led to a re-examination of the molecular networking results to determine if additional features could be tentatively annotated as TMQ-related compounds. After the analysis of the poly-TMQ standard, we expanded the list of diagnostic TMQ fragments to include those related to the TMQ dimer (C_24_H_30_N_2_, [M + H]^+^ = *m*/*z* 347.2488, [M]^+^ = *m*/*z* 346.2403, and fragment [TMQ-C_23_H_26_N_2_ + H]^+^ = *m*/*z* 331.2168, Figure S7). To classify a compound as TMQ-related, at least one of these nine diagnostic MS/MS fragments must be present. When only one diagnostic TMQ fragment was present, a feature was annotated as potentially TMQ-related if connected in the molecular network to another potential TMQ-related feature with two or more diagnostic fragments. As a result, we tentatively annotated 180 TMQ-related features (Table S23). Of particular interest was the presence of TMQ-related features in the 37 aging markers that were found in at least two of the three sample types (Table 2 and Table S20). Nineteen of the 37 aging markers had a molecular weight above 300 Da and were mostly unknown without TMQ-oligomeric information. However, molecular networking allowed the tentative identification of 16 TMQ-related TPs, including 8 of the 37 aging markers of interest (Table 2 and Tables S20 and S24). Six of the TMQ-related TPs have molecular mass >300 and contain oxygen/sulfur atoms. Among these, TP-25, TP-34, TP-35, and TP-36 included one or more TMQ-related oligomers (diagnostic MS/MS fragments: *m*/*z* 346.2403 TMQ dimer, *m*/*z* 331.2168 TMQ-C_23_H_26_N_2_, *m*/*z* 174.1276 TMQ monomer, and TMQ fragment *m*/*z* 158.0967, Figures S8–S11) and benzothiazole analogs (TP-35 and 36 isomers, Figure 5b,c), implying that these higher molecular weight TPs originated from continuous reactions between TMQ-related oligomers and other compounds. While more common environmental transformation pathways such as oxidation may explain some TMQ-related TPs, the presence of TMQ oligomers potentially reacting with other RDCs (e.g., benzothiazoles, Figure 5c) is novel. With growing interest in investigating oligomers as environmental pollutants and toxicants,^82^ further investigation in TMQ, its oligomers, and aging processes is crucial.

**Figure 5:**
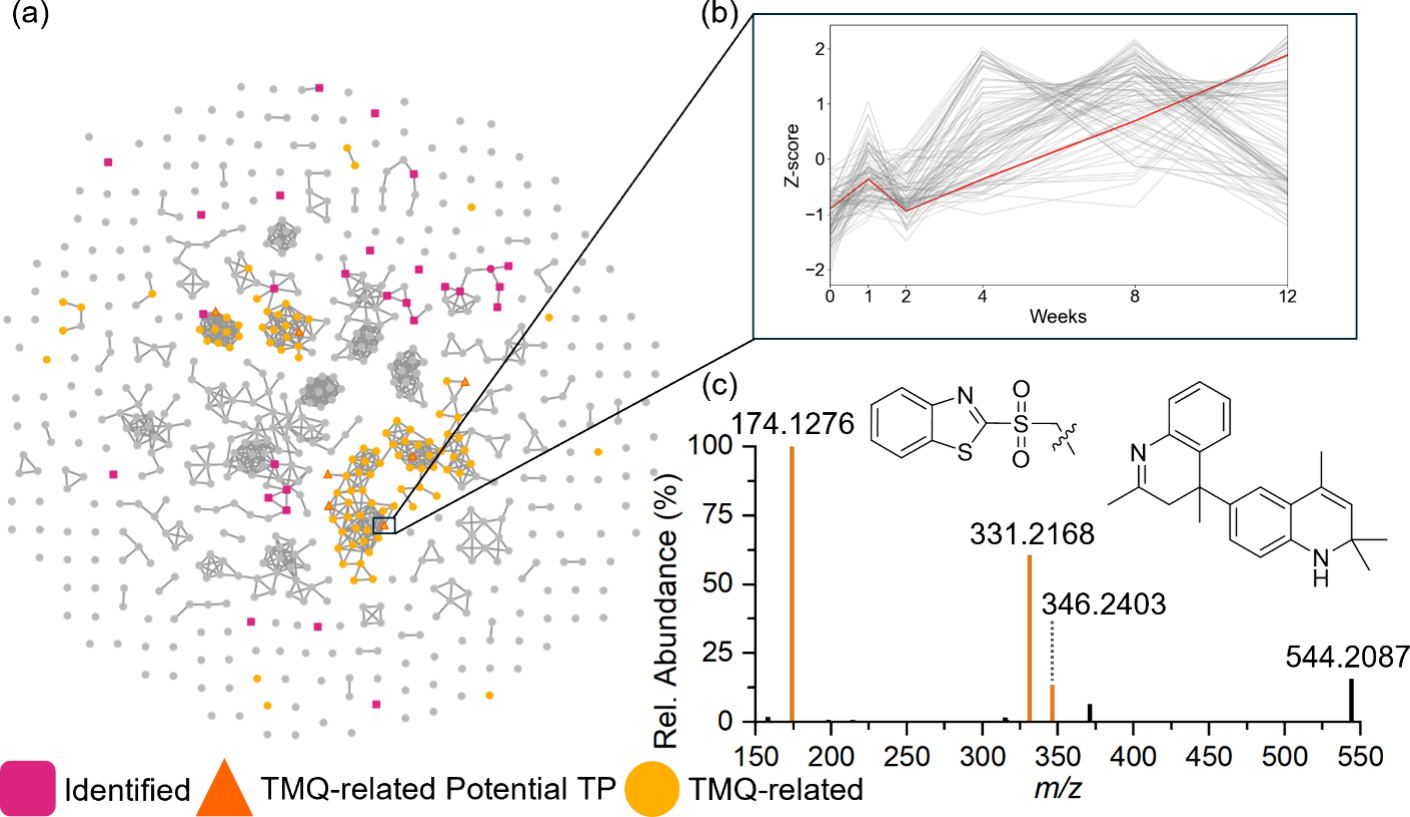
(a) Turf crumb rubber molecular network with TMQ-related features; (b) temporal trend of TP-35; and (c) MS/MS of TP-35 with TMQ diagnostic fragments *m*/*z* 346.2403 (TMQ dimer), *m*/*z* 331.2168 (TMQ-C_23_H_26_N_2_), and *m*/*z* 174.1276 (TMQ monomer) marked in orange.

### 3.3 Limitations and Environmental Implications

While our outdoor aging experiments provide more environmentally relevant conditions than accelerated laboratory studies, the 12-week time frame represents only a fraction of the decades-long environmental persistence of tire materials. Longer-term and field studies on rubber aging will provide further information to help inform the recycling and handling of end-of-life tires. Since all samples were analyzed in ESI positive mode, the results could bias toward N-containing compounds and overlook hydroxylated TPs. The NTS was semiquantitative, so temporal trends might not be conclusive. The faster than predicted degradation rates under outdoor aging suggest the importance of water in aging experiments. The prioritization of 572 potential TPs—of which only ∼63 were tentatively identified—underscores the vast unknown chemical complexity entering environmental systems from the 3 billion tires produced annually.^1^ Our findings on previously detected^81^ but unidentified potential TMQ oligomers and their related high-molecular-weight TPs warrant further investigation given their known bioavailability and potential toxicity. This is especially prevalent from the prioritization of 37 aging markers, of which 8 are TMQ-related. The 37 aging markers provide a benchmark for future research and can guide studies using different tire rubber materials. These markers may reveal which RDCs are most susceptible to transformation, potentially informing rubber manufacturing processes. Additionally, the formation of TPs resulting from reactions of TMQ and other RDCs underscores the need for a deeper understanding of aging and transformation product formation within tire particles. Consequently, current risk assessments on tire rubber and crumb rubber may underestimate TP formation, leaving gaps for exposure monitoring and toxicological studies. The chemical complexity revealed in the rubber aging process suggests that a narrow focus on PPD-related compounds may ignore the more prevalent/abundant contaminants, especially in the context of potential human exposure.

## Supplementary Material





## Data Availability

To support reproducibility and further development, we have made our temporal trend analysis pipeline openly accessible. The source code, along with example data sets and detailed instructions, can be downloaded from https://github.com/YUQIAOTANG/Chemical_compound_for_time_trend_analysis.

## Notes

GNPS workflow parameters (Text S1), kinetic modeling (Text S2), unsupervised temporal trend clustering (Text S3), natural outdoor aging experimental setup (Figure S1), unsupervised temporal trend clusters generated by HDBSCAN for naturally aged TWP wet samples (Figure S2), unsupervised temporal trend clusters generated by HDBSCAN for naturally aged artificial turf crumb rubber wet samples (Figure S3), MS/MS of a monomeric TMQ standard (Figure S4), MS/MS of TMQ-C_21_H_26_N_2_ (Figure S5), MS/MS of TMQ-C_23_H_26_N_2_ (Figure S6), MS/MS of TMQ dimer (C_24_H_30_N_2_) from direct injection of poly-TMQ standard (Figure S7), MS/MS of aging marker TP-25 (TMQ-C_17_H_34_N_2_OS_2_, *m*/*z* 347.2192, RT 19.2) (Figure S8), MS/MS of aging marker TP-34 (TMQ-C_30_H_29_N_3_O_2_S, *m*/*z* 528.1173, RT 18.3) (Figure S9), MS/MS of aging marker TP-35 (TMQ-C31H33N3O2S2, *m*/*z* 544.2085, RT 25.6) (Figure S10), and MS/MS of aging marker TP-36 (TMQ-C31H33N3O2S2, *m*/*z* 544.2087, RT 22.7) (Figure S11) (PDF)
List of standards (Table S1), tire and turf source information (Table S2), natural aging conditions (Table S3), LC-QqQ instrument conditions (Table S4), LC-QqQ transition list (Table S5), LC-q-Orbitrap instrument conditions (Table S6), Skyline settings (Table S7), LOD/LOQ/recovery (Table S8), Compound Discoverer settings (Table S9), GNPS results (Table S10), SIRIUS settings (Table S11), concentrations of target chemicals in all conditions (Table S12), suspect screening results (Table S13), kinetic modeling results from targeted quantitation (Table S14), kinetic modeling results from suspect screening (Table S15), results of unsupervised cluster analysis of CMTT (Table S16), results of unsupervised cluster analysis of TWP (Table S17), results of unsupervised cluster analysis of turf crumb rubber (Table S18), potential transformation products from unsupervised cluster analysis of NTS results (Table S19), aging markers from NTS (Table S20), identified transformation products from NTS (Table S21), diagnostic fragments of unknown features from molecular network (Table S22), list of TMQ-related features from molecular network (Table S23), and identified/annotated TMQ TPs (Table S24) (XLSX)
