# Supplementary material for: From the Road to the Field: Decoding Chemical Transformation in Aging Tire and Artificial Turf Crumb Rubber
Source: Environ Sci Technol. 2025 Dec 18;60(1):1051–62. doi: 10.1021/acs.est.5c08260 (PMC12810383; doi:10.1021/acs.est.5c08260)
Supplement: Supplementary file 1 [file es5c08260_si_001.pdf]

Supplementary Information

From the road to the field: Decoding chemical transformation in aging tire and artificial turf crumb rubber

Madison H. McMinn[a,b], Yuqiao Tang[c,d], Phillip Berger[a,b], Katherine Poisson[a,b], Andresa Oliveira Tavares Lima[a], Aron Stubbins[a,e,f], Arzu Tuğçe Güler[b,d], Zhenyu Tian[a,b *]

[a] Department of Chemistry and Chemical Biology, College of Science, Northeastern University, Boston, MA, 02115, USA

[b] Barnett Institute for Chemical and Biological Analysis, Northeastern University, Boston, MA, 02115, USA

[c] Department of Bioinformatics, College of Science, Northeastern University, Boston, MA, 02115, USA

[d] The Institute for Experiential A.I., Northeastern University, Boston, MA, 02115, USA

[e] Department of Marine and Environmental Sciences, Northeastern University, Boston, MA, 02115, USA

[f] Department of Civil and Environmental Engineering, Northeastern University, Boston, MA, 02115, USA

* Corresponding author

Corresponding author's contact information: z.tian@northeastern.edu

Pages: 15

Figures: 11

Tables: 24

Text S1. GNPS workflow parameters

A molecular network was created using the online workflow (https://ccms-ucsd.github.io/GNPSDocumentation/) on the GNPS website (http://gnps.ucsd.edu).[1] The data was filtered by removing all MS/MS fragment ions within +/- 17 Da of the precursor m/z to remove the residual precursor ion from the MS/MS spectrum used for downstream analysis. MS/MS spectra were window filtered by choosing only the top 6 fragment ions in the +/- 50Da window throughout the spectrum. This is employed to reduce spectral complexity by only keeping the top 6 most intense fragment ions in a +/- 50Da window (e.g., 0-50 Da, 50-100 Da, etc.). The precursor ion mass tolerance was set to 0.02 Da and a MS/MS fragment ion tolerance of 0.02 Da. A network was then created where edges were filtered to have a cosine score above 0.5 and more than 2 matched peaks. Further, edges between two nodes were kept in the network if and only if each of the nodes appeared in each other's respective top 10 most similar nodes. Finally, the maximum size of a molecular family was set to 100, and the lowest scoring edges were removed from molecular families until the molecular family size was below this threshold.

Text S2. Kinetic modeling

For chemicals exhibiting monotonic decay, we employed a first-order exponential model following Fohet et al.[2]:

$$C(t) = C_0 \times e^{-t/\tau} \text{ [Eq. 1]}$$

where $C(t)$ represents the chemical concentration at time t, C_0 denotes the initial concentration, and τ is the characteristic decay time constant.[2]

To characterize compounds displaying non-monotonic behavior, we developed a dual-exponential model that captures both formation and degradation processes:

$$C(t) = C_0 + C_x(-exp^{-t/\tau 1} + exp^{-t/\tau 2}) \text{ [Eq. 2]}$$

Here, τ_1 and τ_2 represent the time constants for formation and dissipation processes respectively, while A is a scaling factor related to the maximum achievable concentration. This formulation extends beyond simple decay models to accommodate transformation products that initially accumulate before degrading.

For chemicals showing progressive accumulation throughout the observation period, we utilized a saturation model following Fohet et al.[2]:

$$C(t) = (C_0 - C_f) \times (exp^{-t/\tau}) + C_f \text{[Eq. 3]}$$

where C_0 and C_f represent the initial and final (asymptotic) concentrations, respectively.

Parameter estimation was performed using nonlinear least-squares regression implemented in R (version 4.4.2). We employed the Levenberg-Marquardt algorithm via the minpack.lm package (version 1.2.4) for robust parameter optimization, with visualization conducted using ggplot2 (version 3.5.2). Model selection was based on visual inspection of residual patterns and the Akaike Information Criterion (AIC) to ensure appropriate fit complexity. Residual patterns were

visually inspected to screen whether the temporal trends were broadly consistent with monotonic models. Systematic deviations in the residuals (for example, a slight initial increase followed by a decline) indicated that more complex models might provide a closer statistical fit. However, such models involve additional parameters that increase the risk of overfitting and yield constants ($C0, Cf, \tau$) of limited interpretive value. So that the AIC was applied, which penalizes unnecessary model complexity. Models with smaller AIC values were favored as a balance between fit quality and parsimony. In borderline cases showing minor non-monotonicity, we classified them as monotonic models, consistent with previous studies using the same formulation for time constant estimation.

Text S3. Unsupervised temporal trend clustering

Unsupervised temporal clustering of NTS data was performed in Python (ver. 3.9, sci-kit learn[3] ver. 1.6.1 , matplotlib[4] ver. 3.9.0) using hierarchical density-based spatial clustering of applications with noise (HDBSCAN[5] ver. 0.8.40). Mean peak areas of chemical features at six time points (0, 1, 2, 4, 8, and 12 weeks) were Z-score normalized to enable pattern-based rather than intensity-based clustering, following standard practices in time series analysis .[6] Initial HDBSCAN clustering parameters were as follows: min_cluster_size = 30, min_samples = 2, cluster_selection_epsilon = 0.8, resulting in three primary groups: noise (-1), cluster (0) and cluster (1).. After the initial generation of these 3 clusters (-1, 0, and 1), manual inspection indicated that cluster (1) required further refinement to separate different temporal trends (increasing, decreasing, and intermediate). Based on t-SNE visualization and distinct degradation/formation kinetics observed within the cluster (1), k-means clustering (k = 4) was subsequently applied to resolve this group into four sub-clusters representing different transformation patterns. The t-SNE projection and temporal profiles were generated for all clusters to facilitate interpretation of compound fate.

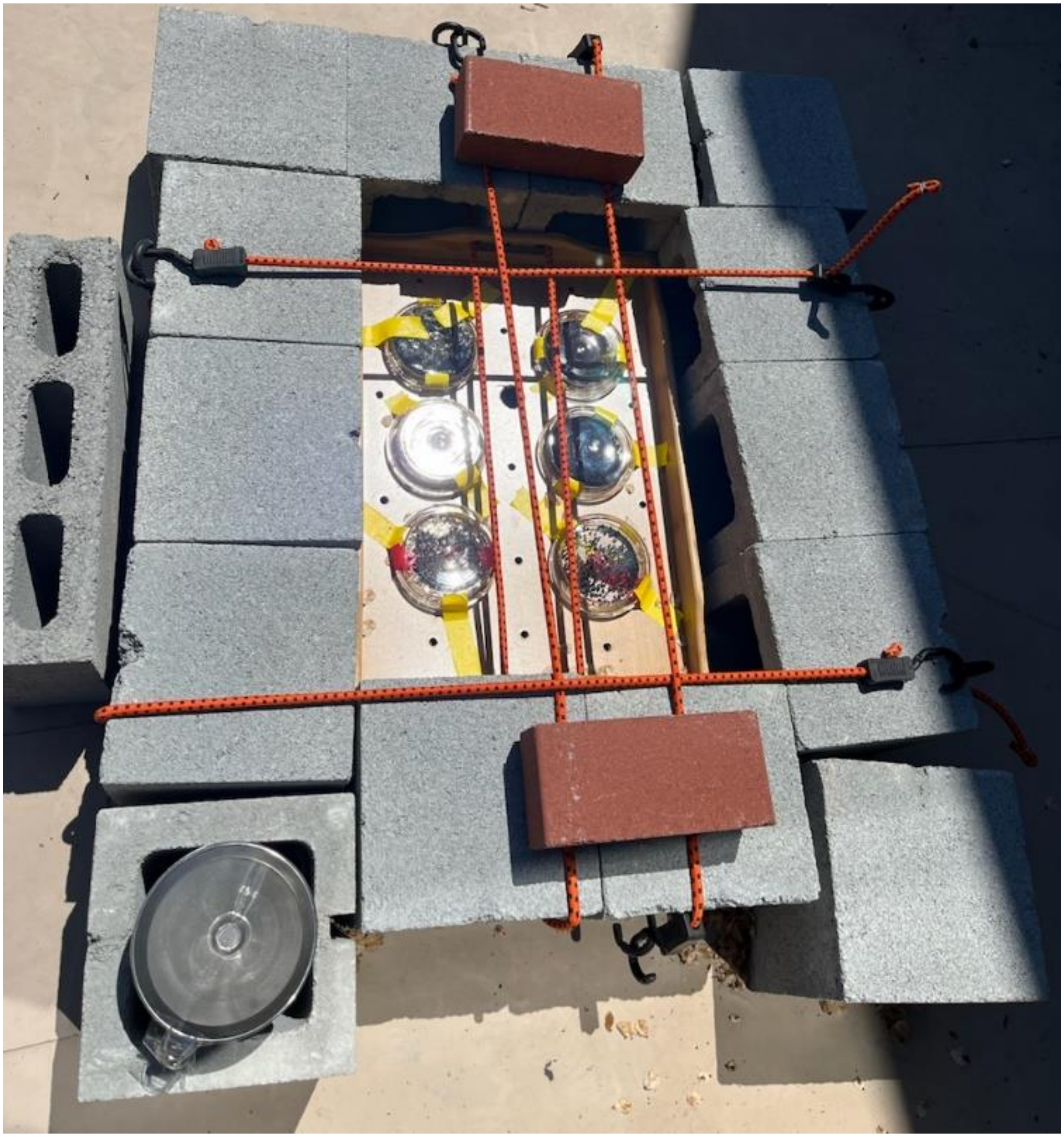

Figure S1. Natural outdoor aging experimental setup.

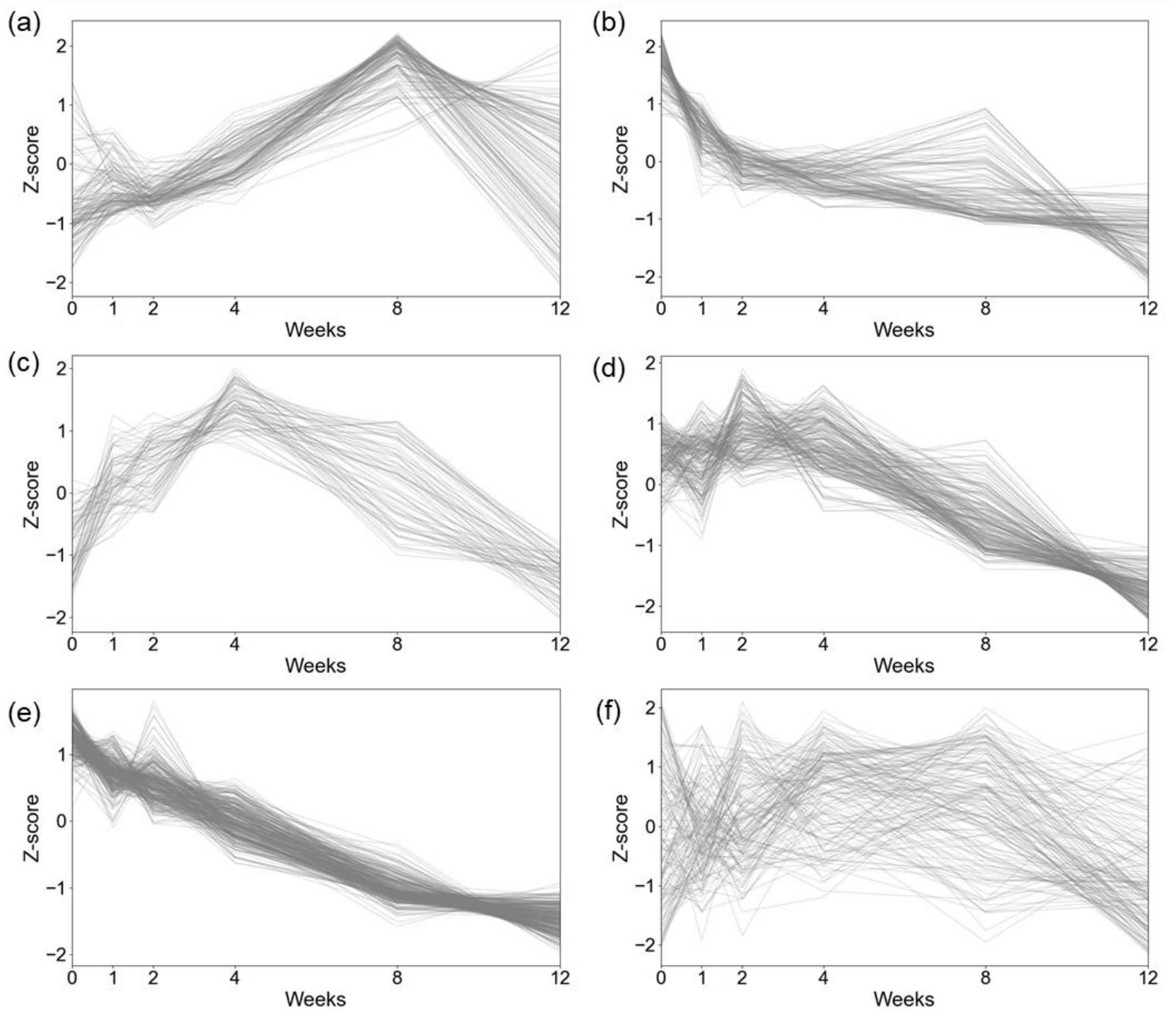

Figure S2. Unsupervised temporal trend clusters generated by HDBSCAN for naturally aged TWP wet samples for (a) cluster #0, intermediate (b) cluster #10, decreasing (c) cluster #11, intermediate (d) cluster #12, decreasing (e) cluster #13, decreasing (f) cluster #-1, no trend.

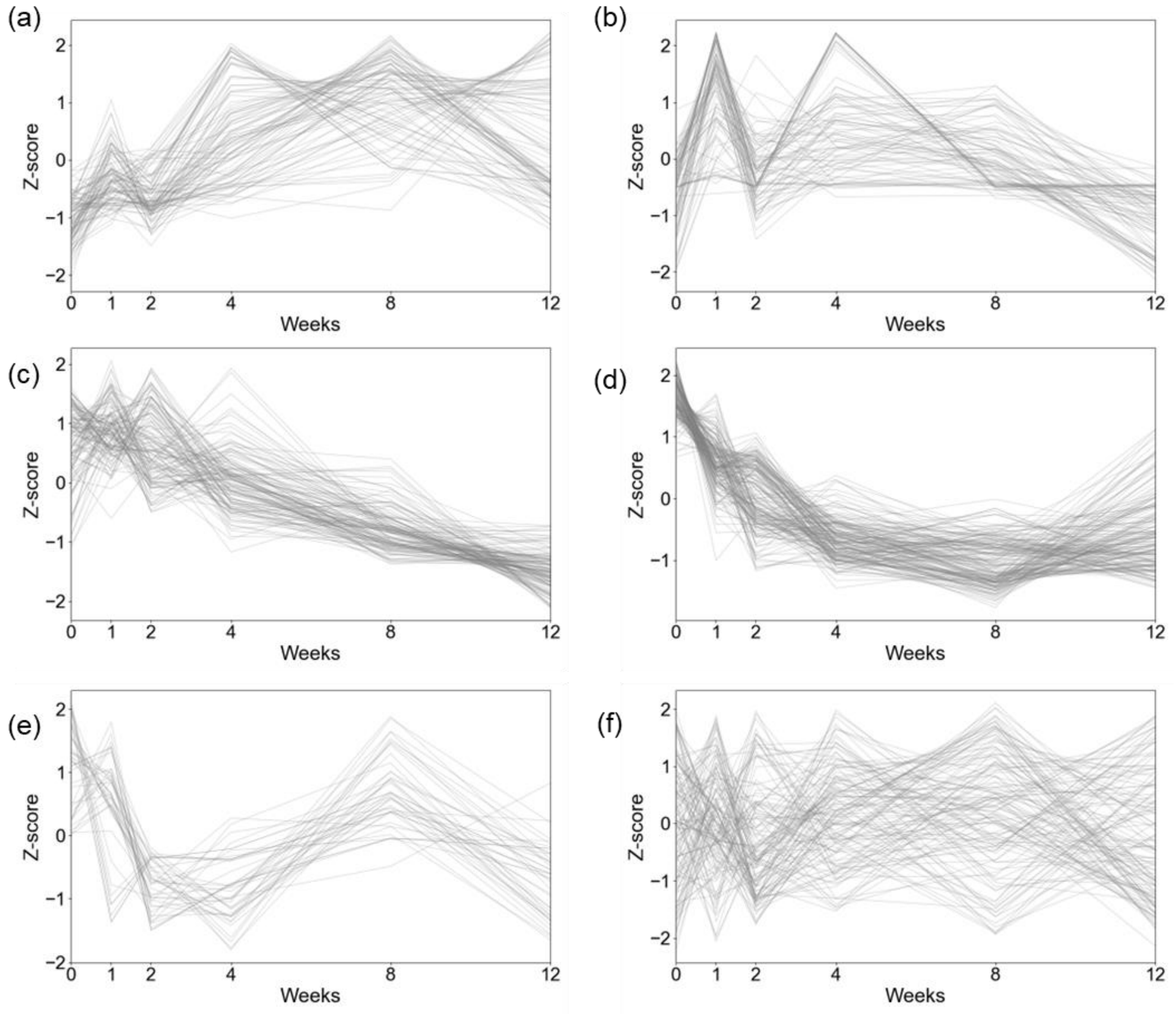

Figure S3. Unsupervised temporal trend clusters generated by HDBSCAN for naturally aged artificial turf crumb rubber wet samples for (a) cluster #0, increasing (b) cluster #10, no trend (c) cluster #11, decreasing (d) cluster #12, decreasing (e) cluster #13, no trend (f) cluster #-1, no trend.

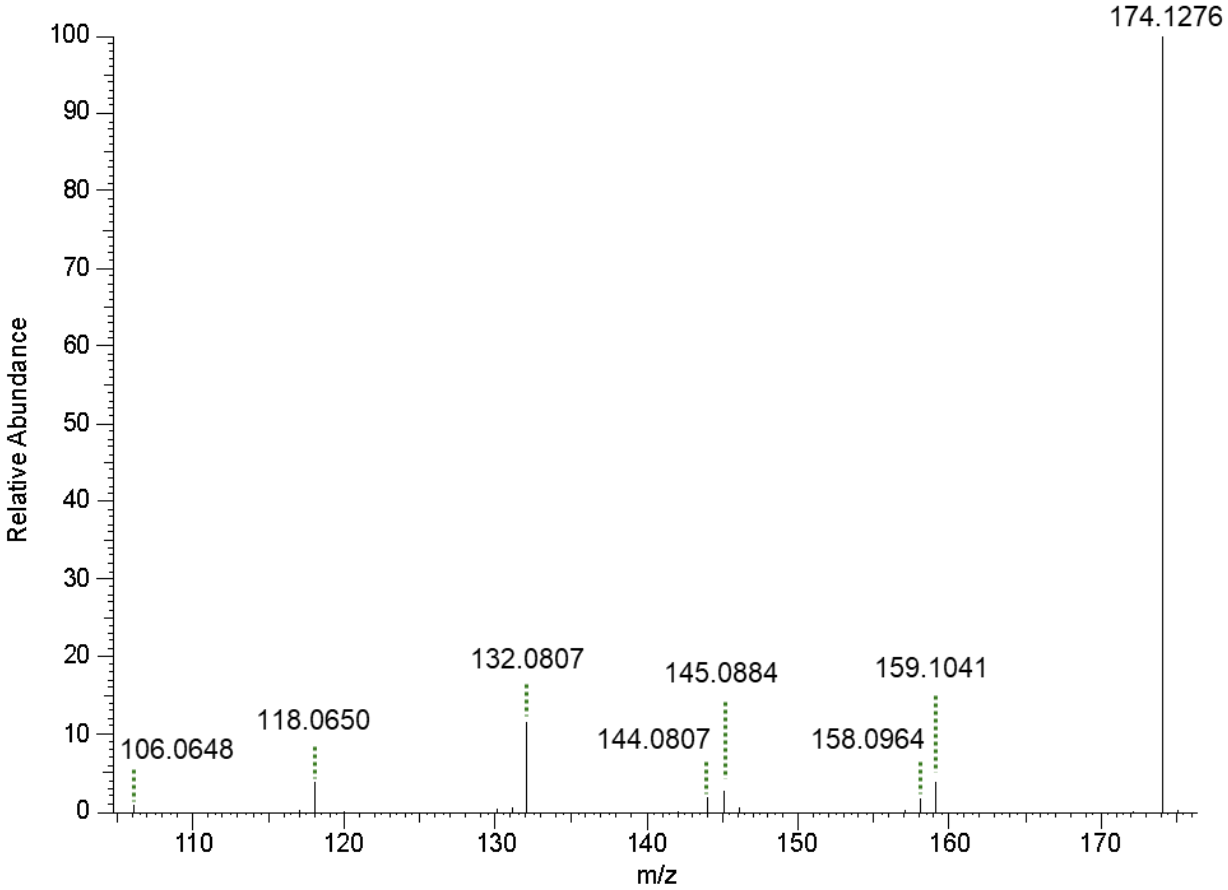

Figure S4. MS/MS of a monomeric TMQ standard.

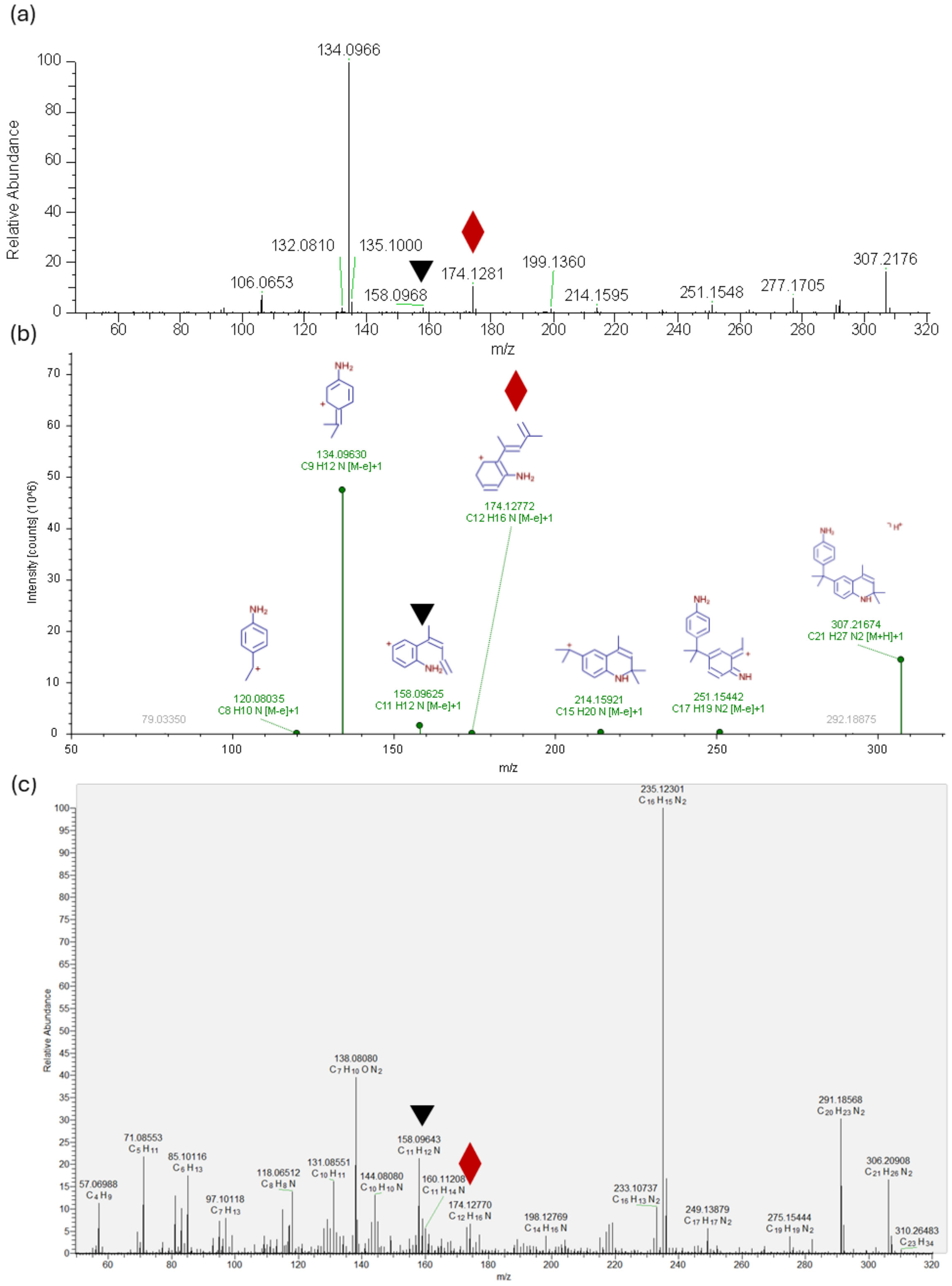

Figure S5. MS/MS of (a) TMQ-$C_{21}H_{26}N_2$ from direct injection of poly-TMQ standard, (b) TMQ-$C_{21}H_{26}N_2$ in turf crumb rubber, and (c) MS/MS from Hagg et al 2023.[7] Diagnostic TMQ fragments found in all samples are annotated.

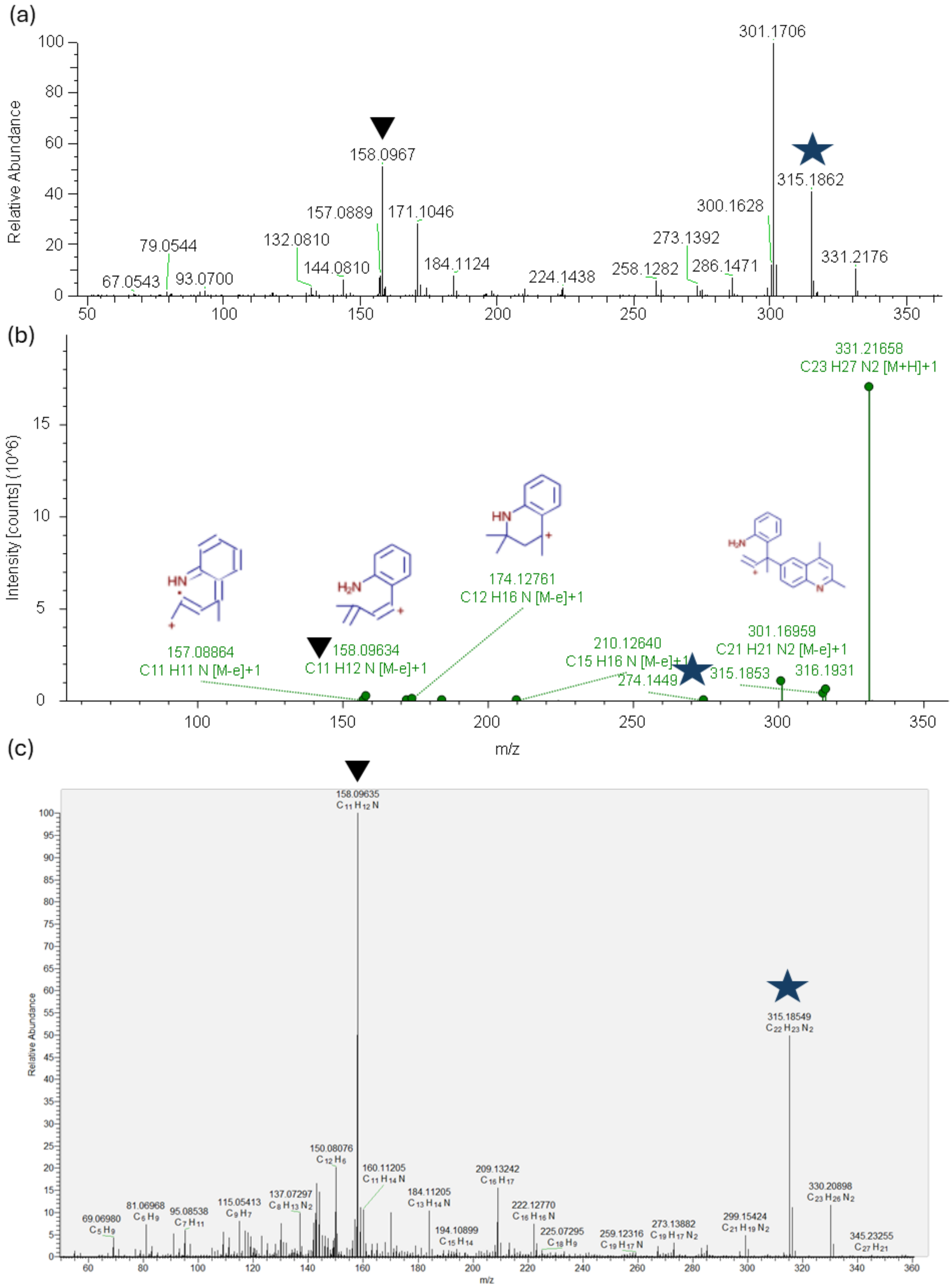

Figure S6. MS/MS of (a) TMQ-$C_{23}H_{26}N_2$ from direct injection of poly-TMQ standard, (b) TMQ-$C_{23}H_{26}N_2$ in turf crumb rubber, and (c) MS/MS from Hagg et al 2023.[7] Diagnostic TMQ fragments found in all samples are annotated.

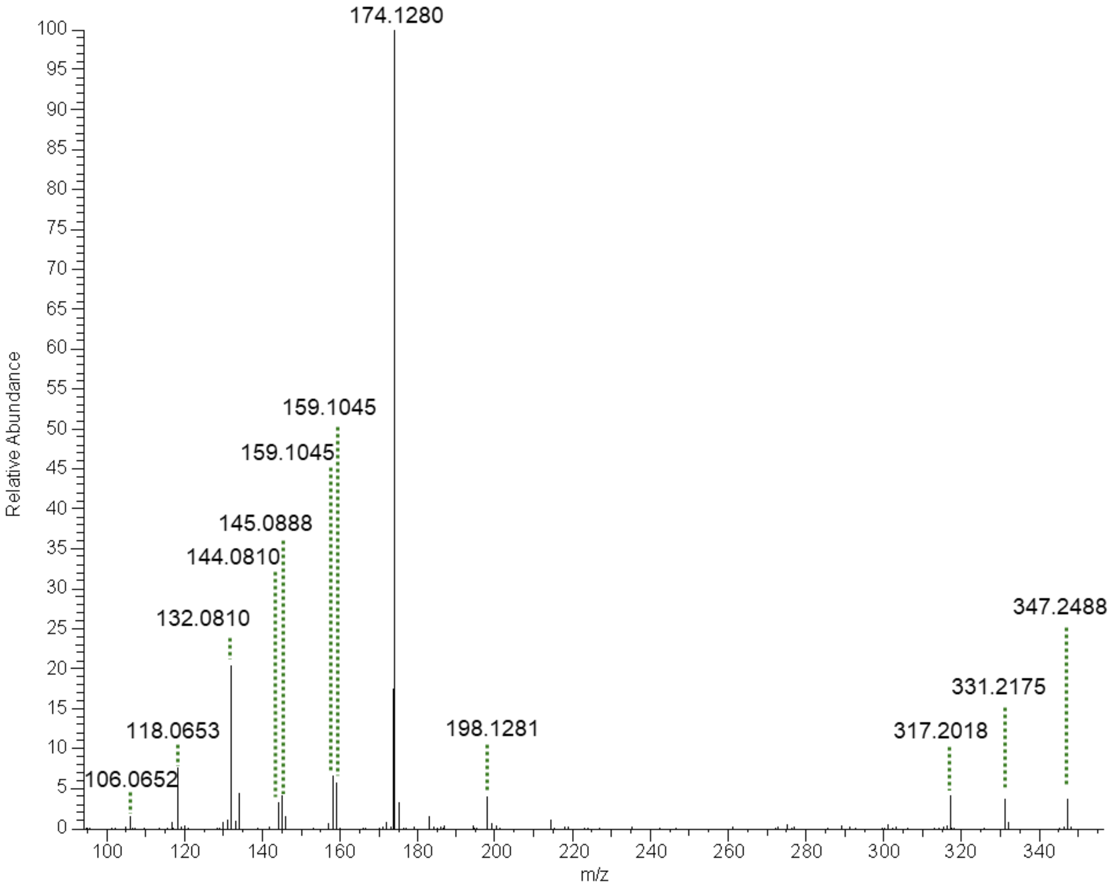

Figure S7. MS/MS of TMQ dimer ($C_{24}H_{30}N_2$) from direct injection of poly-TMQ standard.

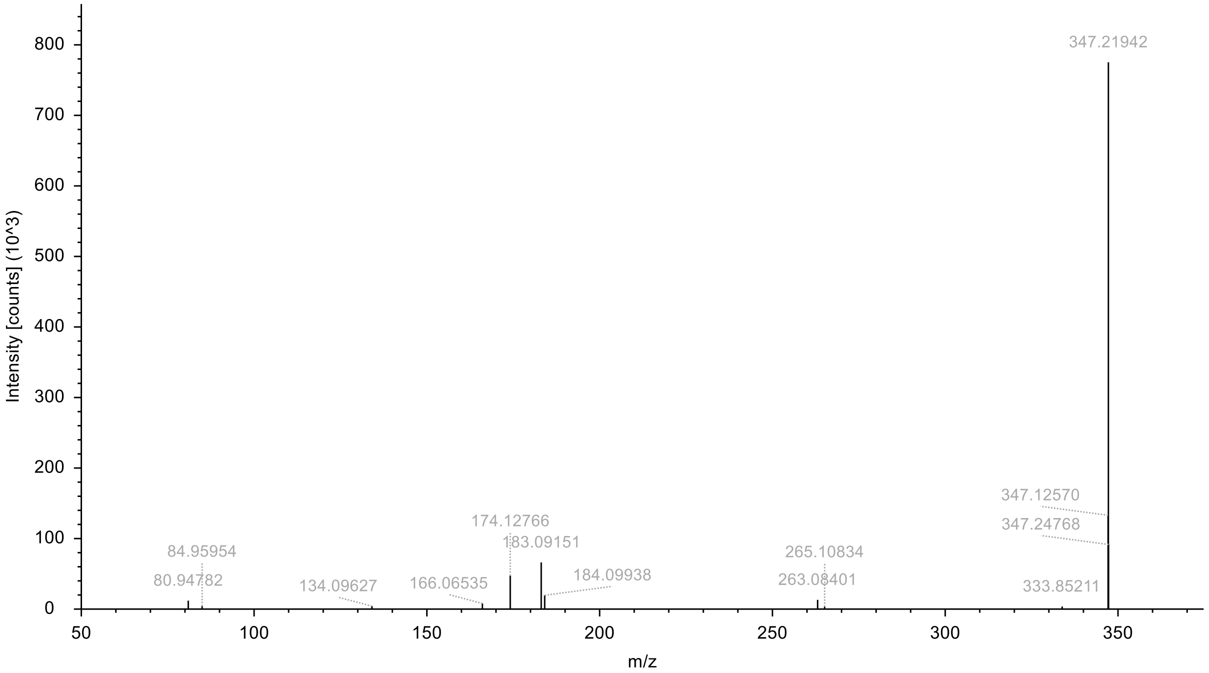

Figure S8. MS/MS of Aging Marker TP25 (TMQ-$C_{17}H_{34}N_2OS_2$, *m/z* 347.2192, RT 19.2).

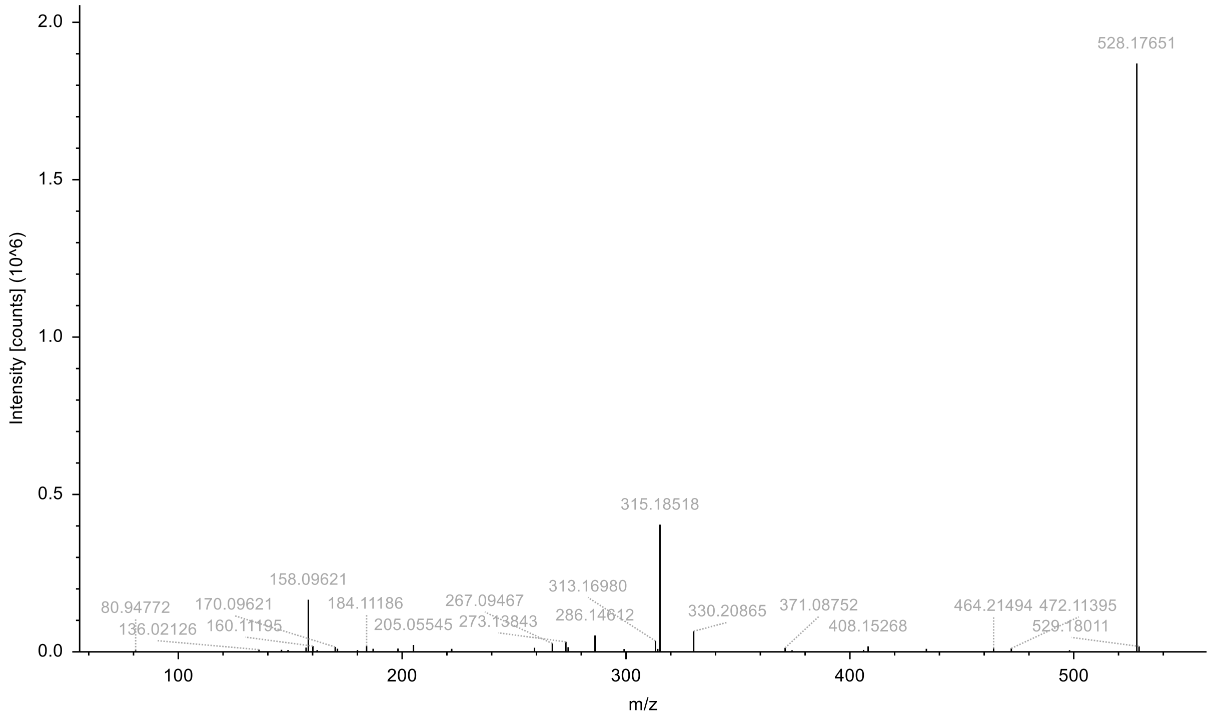

Figure S9. MS/MS of Aging Marker TP34 (TMQ-$C_{30}H_{29}N_3O_2S$, *m/z* 528.1173, RT 18.3).

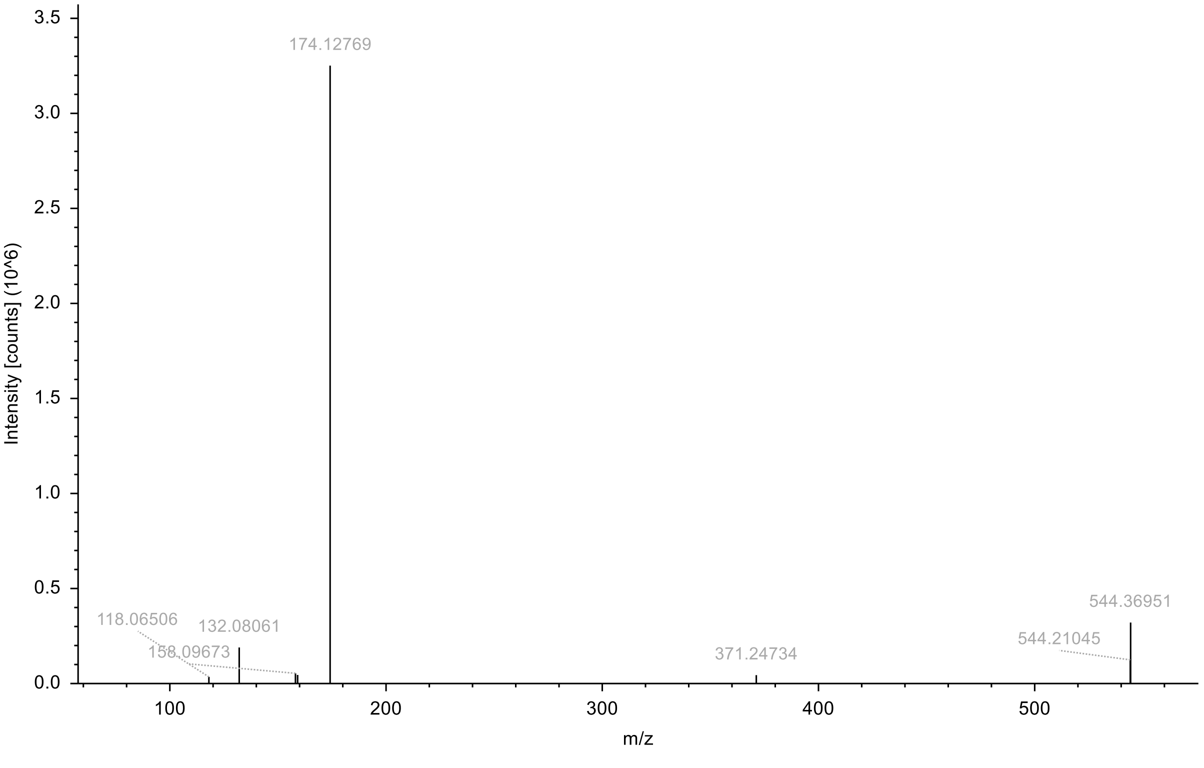

Figure S10. MS/MS of Aging Marker TP35 (TMQ-$C_{31}H_{33}N_3O_2S_2$, *m/z* 544.2085, RT 25.6).

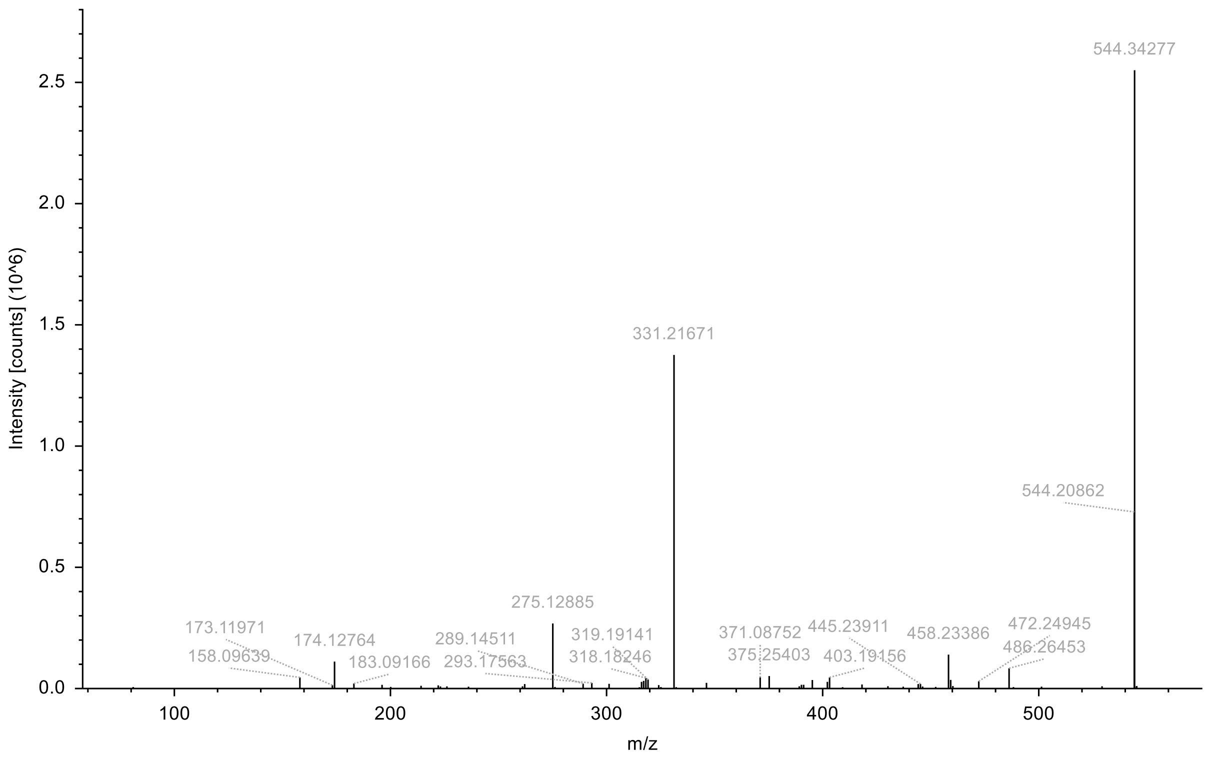

Figure S11. MS/MS of Aging Marker TP36 (TMQ-$C_{31}H_{33}N_3O_2S_2$, *m/z* 544.2087, RT 22.7).

References

(1) Wang, M.; Carver, J. J.; Phelan, V. V.; Sanchez, L. M.; Garg, N.; Peng, Y.; Nguyen, D. D.; Watrous, J.; Kapono, C. A.; Luzzatto-Knaan, T.; Porto, C.; Bouslimani, A.; Melnik, A. V.; Meehan, M. J.; Liu, W. T.; Crusemann, M.; Boudreau, P. D.; Esquenazi, E.; Sandoval-Calderon, M.; Kersten, R. D.; Pace, L. A.; Quinn, R. A.; Duncan, K. R.; Hsu, C. C.; Floros, D. J.; Gavilan, R. G.; Kleigrewe, K.; Northen, T.; Dutton, R. J.; Parrot, D.; Carlson, E. E.; Aigle, B.; Michelsen, C. F.; Jelsbak, L.; Sohlenkamp, C.; Pevzner, P.; Edlund, A.; McLean, J.; Piel, J.; Murphy, B. T.; Gerwick, L.; Liaw, C. C.; Yang, Y. L.; Humpf, H. U.; Maansson, M.; Keyzers, R. A.; Sims, A. C.; Johnson, A. R.; Sidebottom, A. M.; Sedio, B. E.; Klitgaard, A.; Larson, C. B.; P, C. A. B.; Torres-Mendoza, D.; Gonzalez, D. J.; Silva, D. B.; Marques, L. M.; Demarque, D. P.; Pociute, E.; O'Neill, E. C.; Briand, E.; Helfrich, E. J. N.; Granatosky, E. A.; Glukhov, E.; Ryffel, F.; Houson, H.; Mohimani, H.; Kharbush, J. J.; Zeng, Y.; Vorholt, J. A.; Kurita, K. L.; Charusanti, P.; McPhail, K. L.; Nielsen, K. F.; Vuong, L.; Elfeki, M.; Traxler, M. F.; Engene, N.; Koyama, N.; Vining, O. B.; Baric, R.; Silva, R. R.; Mascuch, S. J.; Tomasi, S.; Jenkins, S.; Macherla, V.; Hoffman, T.; Agarwal, V.; Williams, P. G.; Dai, J.; Neupane, R.; Gurr, J.; Rodriguez, A. M. C.; Lamsa, A.; Zhang, C.; Dorrestein, K.; Duggan, B. M.; Almaliti, J.; Allard, P. M.; Phapale, P.; Nothias, L. F.; Alexandrov, T.; Litaudon, M.; Wolfender, J. L.; Kyle, J. E.; Metz, T. O.; Peryea, T.; Nguyen, D. T.; VanLeer, D.; Shinn, P.; Jadhav, A.; Muller, R.; Waters, K. M.; Shi, W.; Liu, X.; Zhang, L.; Knight, R.; Jensen, P. R.; Palsson, B. O.; Pogliano, K.; Linington, R. G.; Gutierrez, M.; Lopes, N. P.; Gerwick, W. H.; Moore, B. S.; Dorrestein, P. C.; Bandeira, N. Sharing and community curation of mass spectrometry data with Global Natural Products Social Molecular Networking. *Nat. Biotechnol.* **2016**, *34* (8), 828-837. DOI: 10.1038/nbt.3597

(2) Fohet, L.; Andanson, J. M.; Charbouillot, T.; Malosse, L.; Leremboure, M.; Delor-Jestin, F.; Verney, V. Time-concentration profiles of tire particle additives and transformation products under natural and artificial aging. *Sci. Total Environ.* **2023**, *859* (Pt 1), 160150. DOI: 10.1016/j.scitotenv.2022.160150

(3) Pedregosa F, V., Ga"el, Gramfort A, Michel V, Thirion B, Grisel O, et al. Scikit-learn: Machine learning in Python. *J. Mach. Learn. Res.* **2011**, *12*, 2825–2830. DOI: 10.5555/1953048.2078195

(4) Hunter, J. D. Matplotlib: A 2D graphics environment. *Computing in Science & Engineering* **2007**, *9* (3), 90-95. DOI: 10.1109/MCSE.2007.55

(5) McInnes, L.; Healy, J.; Astels, S. hdbscan: Hierarchical density based clustering. *J. Open Source Softw.* **2017**, *2* (11), 205. DOI: 10.21105/joss.00205

(6) Lima, F. T.; Souza, V. M. A. A Large Comparison of Normalization Methods on Time Series. *Big Data Research* **2023**, *34*, 100407. DOI: https://doi.org/10.1016/j.bdr.2023.100407

(7) Hägg, F.; Herzke, D.; Nikiforov, V. A.; Booth, A. M.; Sperre, K. H.; Sørensen, L.; Creese, M. E.; Halsband, C. Ingestion of car tire crumb rubber and uptake of associated chemicals by lumpfish (Cyclopterus lumpus). *Front. Environ. Sci.* **2023**, *11*. DOI: 10.3389/fenvs.2023.1219248
